# CD7-deleted hematopoietic stem cells can restore immunity after CAR T cell therapy

**DOI:** 10.1172/jci.insight.149819

**Published:** 2021-08-23

**Authors:** Miriam Y. Kim, Matthew L. Cooper, Miriam T. Jacobs, Julie K. Ritchey, Julia Hollaway, Todd A. Fehniger, John F. DiPersio

**Affiliations:** Department of Medicine, Division of Oncology, Washington University School of Medicine, St. Louis, Missouri, USA.

**Keywords:** Immunology, Oncology, Cancer immunotherapy, Gene therapy, Hematopoietic stem cells

## Abstract

Targeting T cell malignancies with universal CD7-targeting chimeric antigen receptor T cells (UCART7) can lead to profound immune deficiency due to loss of normal T and NK cells. While a small population of endogenous CD7^–^ T cells exists, these cells are unlikely to be able to repopulate the entire immune repertoire after UCART7 treatment, as they are limited in number and proliferative capacity. To rescue T and NK cells after UCART7, we created hematopoietic stem cells genetically deleted for *CD7* (CD7-KO HSCs). CD7-KO HSCs were able to engraft immunodeficient mice and differentiate into T and NK cells lacking CD7 expression. CD7-KO T and NK cells could perform effector functions as robustly as control T and NK cells. Furthermore, CD7-KO T cells were phenotypically and functionally distinct from endogenous CD7^–^ T cells, indicating that CD7-KO T cells can supplement immune functions lacking in CD7^–^ T cells. Mice engrafted with CD7-KO HSCs maintained T and NK cell numbers after UCART7 treatment, while these were significantly decreased in control mice. These studies support the development of CD7-KO HSCs to augment host immunity in patients with T cell malignancies after UCART7 treatment.

## Graphical abstract

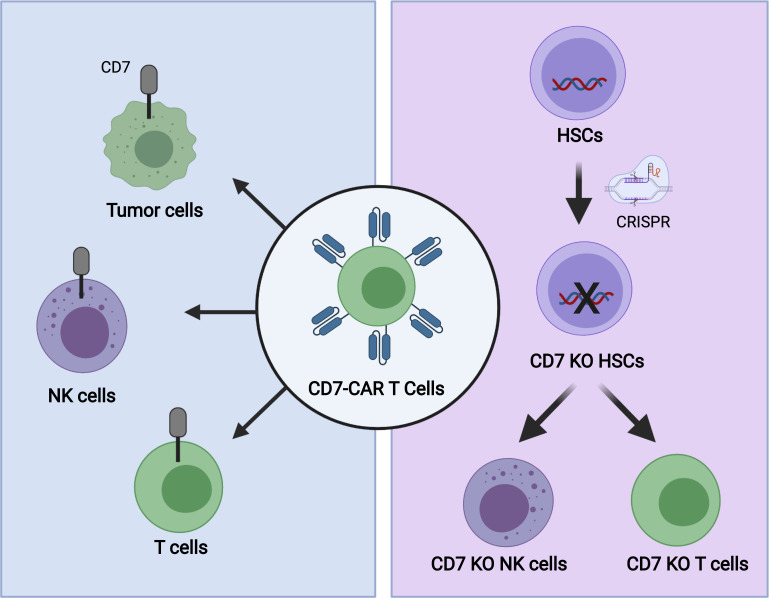

## Introduction

Chimeric antigen receptor (CAR) T cells targeting CD19 are an effective therapy for B cell malignancies. However, expanding the reach of this treatment beyond B cell malignancies is challenging due to the paucity of antigens that can be safely targeted without causing undue toxicity. One strategy to circumvent this problem is to genetically delete the target antigen from hematopoietic stem cells (HSCs), which can then generate an antigen-negative hematopoietic system that is resistant to CAR T cell therapy. As proof of principle, we and others have shown that CD33, a myeloid antigen, can be safely deleted from HSCs and can generate CD33^–^ myeloid cells that are resistant to CD33-targeting immunotherapy including CAR T cells (1–3).

We now propose to extend this strategy to T cell antigens. T cell malignancies pose an additional challenge to CAR T cell therapy due to the shared expression of target antigens between normal and malignant T cells. To overcome this problem, our group has developed CAR T cells targeting CD7 that are genetically edited to lack both CD7 and the T cell receptor (universal CD7-targeting chimeric antigen receptor T cells [UCART7]) in order to avoid fratricide and permit the use of allogeneic T cells for treatment (4). Other groups have also independently developed strategies to mitigate fratricide by removing CD7 expression from CAR T cells (5, 6). However, none of these strategies address the limitation of toxicity to normal T and NK cells that may result in sustained immunodeficiency and susceptibility to opportunistic infections.

Here, we show that eliminating all CD7-expressing cells would be detrimental to human health, as this would eliminate most of the cytotoxic effector cells of the immune system, and the remaining CD7^–^ T cells are not capable of the full spectrum of immune cell function. We then show that genetic deletion of the *CD7* gene in HSCs is feasible without impairing their ability to engraft and differentiate into mature hematopoietic cells, including T and NK cells. Furthermore, we find that CD7-KO T cells are functionally indistinguishable from control T cells and retain properties of CD7^+^ T cells that are lacking in CD7^–^ T cells. Finally, we show that CD7-KO T and NK cells are resistant to UCART7 attack and, thus, can preserve host immunity after CAR T cell treatment.

## Results

### Characterization of CD7^–^ T cells.

**Figure 1:**
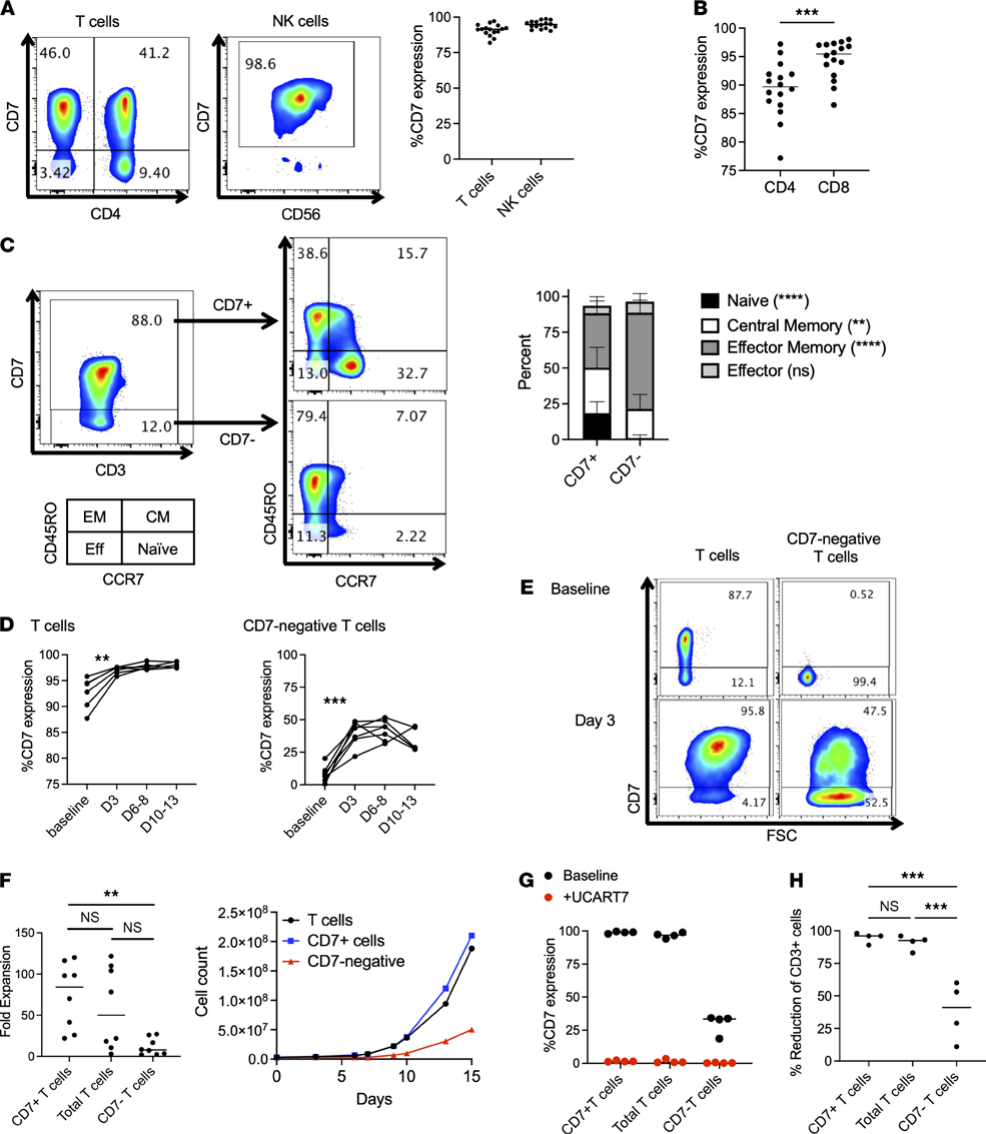
CD7^–^ T cells have limited proliferative capabilities and are potentially still susceptible to UCART7 after activation. (**A**–**C**) CD7 expression on healthy T and NK cells was measured by flow cytometry (*n* = 16 donors). (**A**) CD7 expression on T cells and NK cells. (**B**) CD7 expression on CD4^+^ and CD8^+^ T cell subsets (paired 2-tailed Student’s *t* test). (**C**) CD7^–^ T cells exhibit a predominantly effector memory phenotype. Memory phenotype was defined by CD45RO and CCR7 expression, as depicted in the left panel. Asterisks denote statistical comparisons of the frequency of each group within CD7^+^ versus CD7^–^ cells by 2-way ANOVA with multiple comparisons. (**D**–**F**) T cells were divided into CD7^+^ and CD7^–^ fractions and activated with anti-CD3/CD28 beads, followed by serial cell counts and flow cytometry to evaluate CD7 expression (*n* = 8 donors). (**D**) CD7 expression significantly increases after T cell activation with anti-CD3/CD28 beads in both bulk T cells (left) and CD7^–^ cells (right) (paired 2-tailed Student’s *t* test). (**E**) Representative flow cytometry plots showing increased CD7 expression in total and CD7^–^ T cells after activation. (**F**) Maximum fold-expansion of CD7^+^, total, and CD7^–^ T cells (1-way ANOVA with Tukey’s multiple-comparison test). Representative growth curve is shown on the right. (**G** and **H**) Activated T cells were incubated with UCART7 for 48 hours, after which cell counts and percentage of CD7 of remaining CD3^+^ T cells were quantified by flow cytometry (*n* = 4 donors). All numbers are averages of 3 technical replicates. (**G**) Percentage of CD7 expression on CD3^+^ T cells at baseline and after incubation with UCART7. (**H**) Percent reduction of CD3^+^ T cells in each group after UCART7 treatment, calculated as the number of CD3^+^ cells in UCART7-treated groups divided by the number of CD3^+^ cells in untreated groups (1-way ANOVA with Tukey’s multiple-comparison test). **P < 0.01, ***P < 0.001, ****P < 0.0001.

To confirm the previously reported expression pattern of CD7 (7, 8), we evaluated CD7 expression in healthy donor peripheral blood mononuclear cells (PBMCs). Consistent with prior reports, we found that CD7 was expressed in the majority of T cells (median 90%) and NK cells (median 97%) (Figure 1A). There was a discernable CD7^–^ T cell population in all subjects that was slightly more prominent in CD4^+^ cells (Figure 1B). Immunophenotyping by FACS showed decreased CCR7 and increased CD45RO expression in the CD7^–^ T cell population, so that the majority of cells had an effector memory phenotype (Figure 1C). Of note, these cells were obtained from adult donors across a broad age spectrum (range, 22–73 years; median age, 54). T cells from cord blood were predominantly naive, as expected, and uniformly expressed CD7 (Supplemental Figure 1A; supplemental material available online with this article; https://doi.org/10.1172/jci.insight.149819DS1).

To further profile CD7^–^ T cells, we explored publicly available single-cell RNA sequencing (RNA-seq) data sets of healthy donor PBMCs (9–11). We found that CD7 transcript levels in T cells (defined as cells expressing *TRAC* and *CD3E*) in younger individuals (6–16 years) were significantly higher than older individuals (approximately 20–80 years), while T cells from supercentenarians (>110 years) had much lower expression of CD7 than the other 2 age cohorts (Supplemental Figure 1B). We were also able to verify that CD7 expression was higher in CD8^+^ T cells than in CD4^+^ T cells (Supplemental Figure 1C). Differential gene expression analysis of CD7^–^ versus CD7^+^ T cells confirmed a small but significant decrease in expression of *CCR7* and *SELL*, and showed increased expression of effector molecules such as *GZMA* and *GZMH* (Supplemental Figure 1D).

In terms of functionality, CD7^–^ T cells were for the most part intact in their ability to respond to PMA and IL-2, and to degranulate and produce cytokines (Supplemental Figure 2, A–C). However, we noted that CD7 expression increased in T cells after activation with CD3/CD28 beads, from median expression levels at baseline of 92.6%–97% after activation (*P* = 0.0056), suggesting that either CD7^–^ T cells express CD7 upon activation or CD7^–^ T cells are at a proliferative disadvantage compared with CD7^+^ T cells. To investigate this further, we separated CD7^+^ and CD7^–^ T cell populations and activated them separately, and we found that, indeed, a proportion of CD7^–^ T cells expressed CD7 after activation, from median expression of 8.1% at baseline to 39.6% after activation (*P* = 0.0002) (Figure 1, D and E). Additionally, we found that the growth of CD7^–^ T cells was greatly diminished as compared with CD7^+^ T cells (median 8-fold versus 56-fold, *P* = 0.02) (Figure 1F).

To further interrogate T cell function, we generated CAR T cells independently from CD7^+^ and CD7^–^ T cell fractions, in addition to the bulk T cell population. CD7^–^ T cells could be manufactured into CD19- and CD33-targeting CAR T cells that were able to kill tumor cells expressing the target antigen (Supplemental Figure 2, D and E). However, the expansion of CAR T cells generated from CD7^–^ T cells was greatly decreased as compared with bulk T cells or CD7^+^ T cells (Supplemental Figure 2, F and G), and CD7 expression was upregulated on the CAR T cells during expansion (Supplemental Figure 2H).

To assess the susceptibility of the different T cell populations to CD7-targeting therapy, we incubated activated total T cells, CD7^+^ T cells, and CD7^–^ T cells with UCART7. We observed complete elimination of CD7-expressing cells in all groups (Figure 1G), leading to loss of greater than 90% of total and CD7^+^ T cells (Figure 1H), as expected. CD7^–^ T cell numbers were also reduced (range, 11%–60%), confirming that activated CD7^–^ T cells can become susceptible to UCART7, leading to further reduction of T cell numbers after this treatment.

### CD7-KO HSCs can generate CD7-KO T and NK cells.

**Figure 2:**
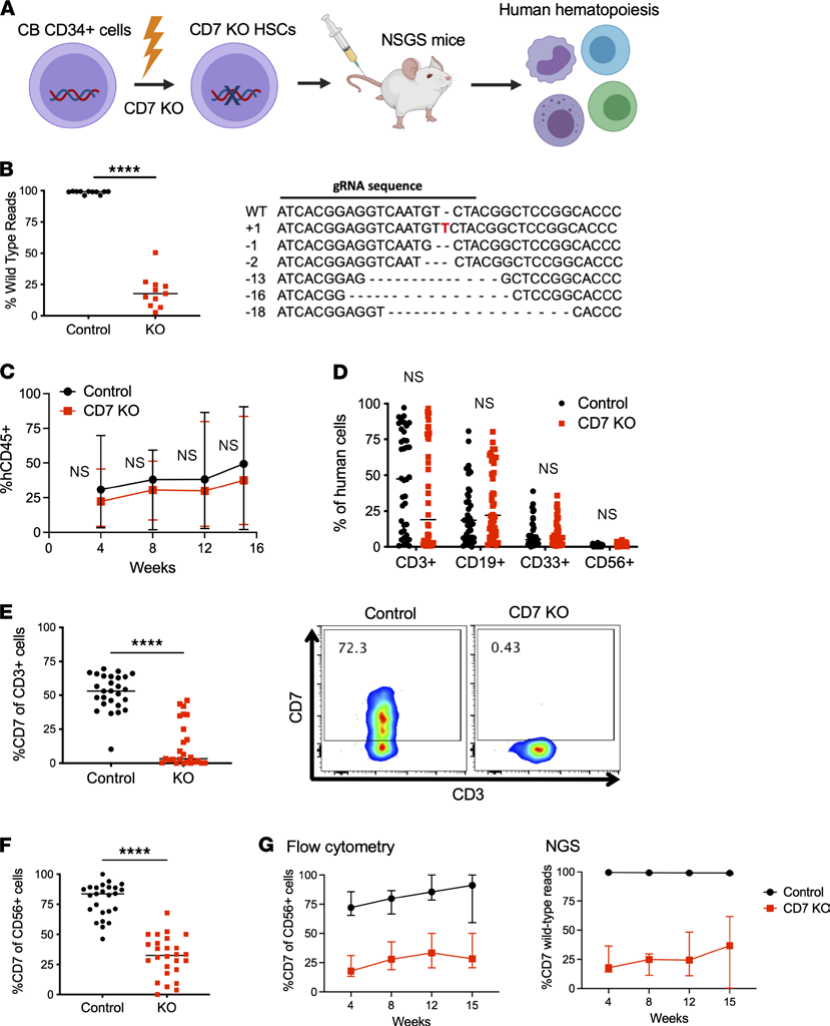
CD7-KO HSCs can engraft and differentiate into T and NK cells lacking CD7 expression. (**A**) Experimental schema: Cord blood CD34^+^ cells were subject to CRISPR/Cas9 editing of the *CD7* gene followed by injection into NSGS mice to assess for their ability to regenerate human hematopoiesis. (**B**) Next-generation sequencing of the *CD7* gene in control and CD7-KO HSCs after gene editing and in vitro differentiation (*n* = 11 cord blood donors). (**C**) Serial measurements of peripheral blood human engraftment in NSGS mice injected with control or CD7-KO HSCs. (**D**) Peripheral blood human T cells (CD3^+^), B cells (CD19^+^), myeloid cells (CD33^+^), and NK cells (CD56^+^) measured at 14–16 weeks (*n* = 42 mice/group). (**E**) CD7 expression in T cells derived from control or CD7-KO HSCs in the peripheral blood (*n* = 25–27 mice/group) (left). Representative flow cytometry plots (right). (**F**) CD7 expression in NK cells derived from control or CD7-KO HSCs in the peripheral blood (*n* = 25–26/group) (**G**) CD7 expression on human NK cells measured by flow cytometry (left) and peripheral blood total *CD7* gene mutations detected by NGS (right) (*n* = 7 mice/group, representative of 3 independent experiments). All statistical analyses were performed using unpaired 2-tailed Student’s *t* test. ****P < 0.0001.

We hypothesized that genetically deleting *CD7* in HSCs could generate CD7^–^ T and NK cells that would provide immune protection to patients undergoing UCART7 treatment. We generated CD7-KO HSCs from human umbilical cord blood CD34^+^ cells using CRISPR/Cas9 (Figure 2A), using the same guide RNA (gRNA) that we previously utilized to delete CD7 in T cells (4, 12). Next-generation sequencing (NGS) showed a median indel frequency of 84% at the *CD7* gene locus, which is comparable with our prior experience when editing T cells (Figure 2B). NSGS mice injected with control or CD7-KO HSCs showed similar levels of human cell engraftment, with differentiation into both myeloid and lymphoid lineages (Figure 2, C and D). Importantly, we found that T cells derived from CD7-KO HSCs showed significantly lower levels of CD7 expression as compared with controls (Figure 2E). CD7 expression was also significantly reduced in the NK cells that emerged in CD7-KO HSC–engrafted mice (Figure 2F), and serial measurements of CD7 surface protein expression in NK cells by flow cytometry correlated with degree of *CD7* gene mutations detected by NGS (Figure 2G).

To better illuminate the engraftment and function of NK cells, we performed these same studies in NSG–huIL-15 mice (13). NSG–huIL-15 mice engrafted with control or CD7-KO HSCs had similar levels of human cell engraftment (Supplemental Figure 3A), and robust human NK cell engraftment was seen with both control and CD7-KO HSCs (Supplemental Figure 3B). We also confirmed that CD7 expression was markedly decreased in NK cells after CD7-KO by both flow cytometry and NGS (Supplemental Figure 3, C and D).

To evaluate whether loss of CD7 confers any growth advantage or disadvantage in hematopoietic cells, we compared the frequency of mutations detected in the infused CD34^+^ cell product and in vivo differentiated human cells after 16 weeks, and we found that the overall frequency of *CD7* gene mutations remained stable (Supplemental Figure 4A). To fully interrogate the regenerative potential of CD7-KO HSCs, we injected control or CD7-KO HSCs into a cohort of NSG mice and confirmed human engraftment (Supplemental Figure 4B), and we then performed secondary transplants into NSGS mice. We found that CD7-KO HSCs retained their ability to engraft and differentiate into human T cells in the secondary recipients, with sustained loss of CD7 expression (Supplemental Figure 4, C–E).

### CD7-KO T and NK cells are functional.

**Figure 3:**
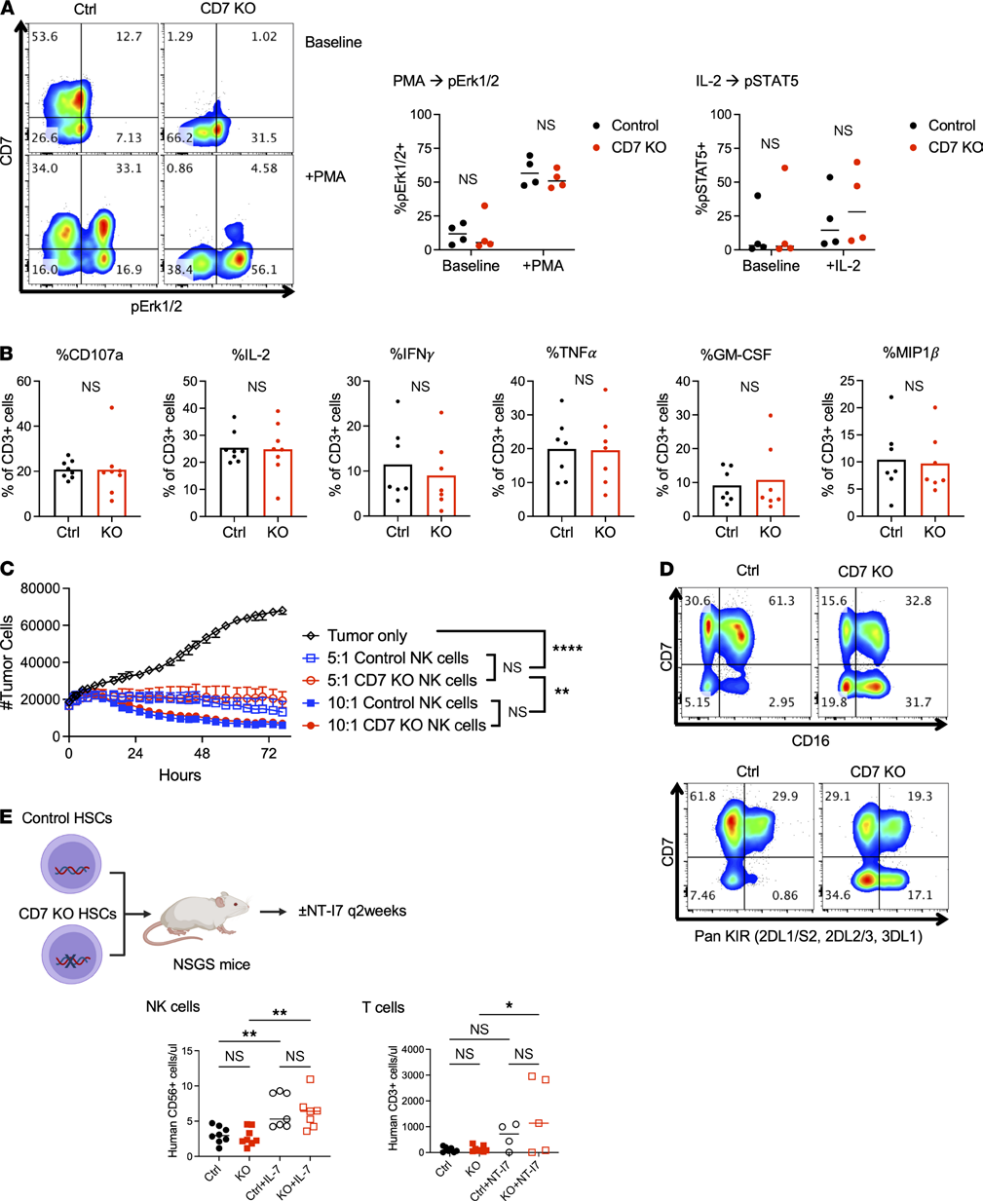
CD7-KO T and NK cells remain functional. (**A** and **B**) Human T cells in the spleens of NSGS mice engrafted with control or CD7-KO HSCs were functionally interrogated in vitro. (**A**) Levels of pErk1/2 and pSTAT5 were assessed in control and CD7-KO T cells at baseline and in response PMA and IL-2 stimulation (*n* = 4 mice/group) (unpaired 2-tailed Student’s *t* test). (**B**) Degranulation (%CD107a) and intracellular cytokine production (%IL-2, IFN-γ, GM-CSF, TNF-α, MIP1b) in control and CD7-KO T cells was measured after stimulation with PMA and ionomycin. (*n* = 7 mice/group) (unpaired 2-tailed Student’s *t* test). (**C** and **D**) Human NK cells purified from the spleens of NSG–huIL-15 mice engrafted with control or CD7-KO HSCs were interrogated in vitro. (**C**) Control and CD7-KO NK cells were incubated at different E:T ratios with UM-SCC-9, a human squamous cell carcinoma cell line, and tumor cell numbers were serially measured over time using the Incucyte Live Cell Analysis system (data expressed as median range of triplicate wells) (2-way ANOVA with multiple comparisons). (**D**) Expression of CD16 and KIR was measured in control and CD7-KO NK cells. (**E**) Mice engrafted with control or CD7-KO HSCs were treated with NT-I7, a long-acting IL-7 agonist, s.c. every 2 weeks starting 1 week after HSC injection for 7 doses total (*n* = 7–8 mice/group). Numbers of peripheral blood human NK cells and T cells are shown at 4 weeks and 15 weeks, respectively, after HSC engraftment (1-way ANOVA with Holm-Sidak multiple-comparison test). *P < 0.05, **P < 0.01, ****P < 0.0001.

To determine whether CD7-KO T cells were functionally intact, we interrogated human T cells obtained from control or CD7-KO HSC–engrafted NSGS mice. Control and CD7-KO T cells both demonstrated appropriate activation of intracellular signaling pathways in response to PMA and IL-2 (Figure 3A). Degranulation (%CD107a) and intracellular cytokine production (%IL-2, IFN-γ, TNF-α, GM-CSF, MIP1b) in response to PMA/ionomycin was also intact in CD7-KO T cells (Figure 3B). To assess the functionality of CD7-KO NK cells, human NK cells obtained from NSG–huIL-15 mice were cultured with UM-SCC-9, a squamous cell carcinoma cell line with known susceptibility to NK cell–mediated cytotoxicity. We found that treatment with control or CD7-KO NK cells led to equivalent tumor cell killing (Figure 3C). Levels of CD16 and killer cell Ig-like receptor (KIR) expression was also comparable in control and CD7-KO NK cells (Figure 3D). To assess the ability of lymphoid cells to respond to cytokine stimulation in vivo, we administered NT-I7, a long-acting recombinant human IL-7 (14, 15), into NSGS mice engrafted with control or CD7-KO HSCs. As expected, levels of circulating human NK cell and T cell numbers increased after administration of NT-I7, and the absence of CD7 made no difference in the degree of response (Figure 3E).

### CD7-KO T cells are distinct from CD7^–^ T cells.

**Figure 4:**
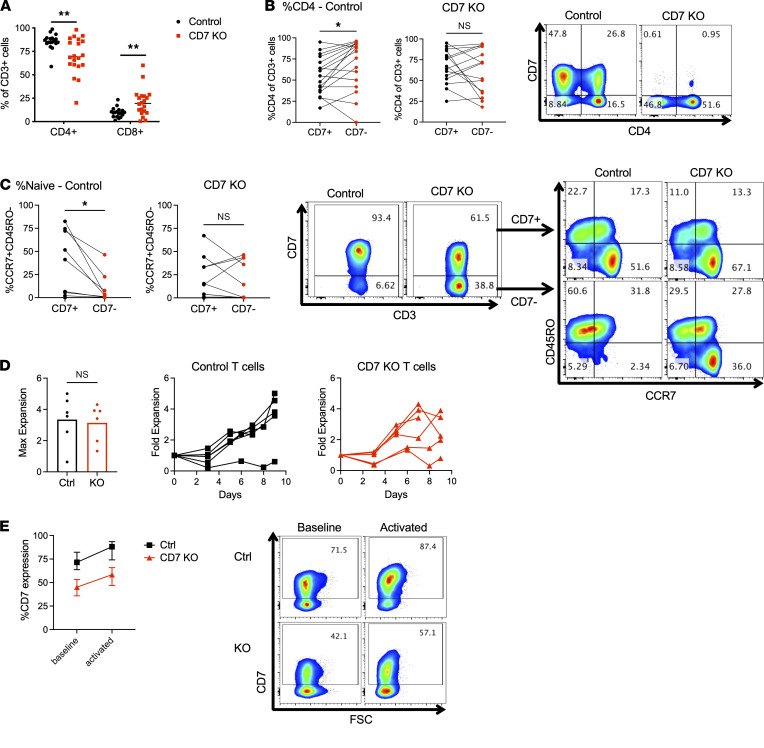
CD7-KO T cells are phenotypically and functionally distinct from CD7^–^ T cells. (**A**) CD4 and CD8 expression on T cells generated from control or CD7-KO HSCs engrafted in NSGS mice (*n* = 22–24/group). (**B**) CD4 expression on CD7^+^ or CD7^–^ T cells derived from control or CD7-KO HSCs (*n* = 15/group). (**C**) Naive cells (CCR7^+^CD45RO^–^) within the CD7^+^ and CD7^–^ T cell fractions in control or CD7-KO T cells (*n* = 10/group). (**D**) Human T cells derived from control or CD7-KO HSCs were expanded in vitro with anti-CD3/CD28 beads (*n* = 6/group) (**E**) CD7 expression at baseline and 3 days after activation in control and CD7-KO T cells (*n* = 5/group). Statistical analyses were performed using unpaired 2-tailed Student’s *t* test (**A** and **D**) or paired Student’s *t* test (**B** and **C**). *P < 0.05, **P < 0.01.

We found that T cells derived from CD7-KO HSCs in NSGS mice had a slight decrease in the percentage of CD4 (%CD4) and a commensurate increase in %CD8 expression that only manifested after several experiments were pooled (control, *n* = 22 mice; CD7-KO, *n* = 24 mice) (Figure 4A). Detailed examination of the CD7^+^ and CD7^–^ T cell fractions in each group revealed that the control T cells had increased CD4 expression in the CD7^–^ fraction, as seen in normal human T cells; however, in CD7-KO T cells this effect was lost (Figure 4B). Therefore, even though CD7-KO T cells had less CD7expression, there was increased expression of CD8 as compared with control T cells. Additionally, while control T cells had the expected decrease in naive cells in the CD7^–^ fraction, CD7-KO T cells retained a naive population within the CD7^–^ cells (Figure 4C). To measure the expansion capacity of CD7-KO T cells, we stimulated control or CD7-KO T cells with CD3/CD28 beads and found no significant difference in expansion between the 2 groups (Figure 4D). Additionally, while both control and CD7-KO T cells upregulated CD7 expression after activation, the absolute difference in CD7 expression was unchanged, indicating that a static population of T cells unable to express CD7 remained in the CD7-KO group (Figure 4E).

### CD7-KO T and NK cells are resistant to UCART7.

**Figure 5:**
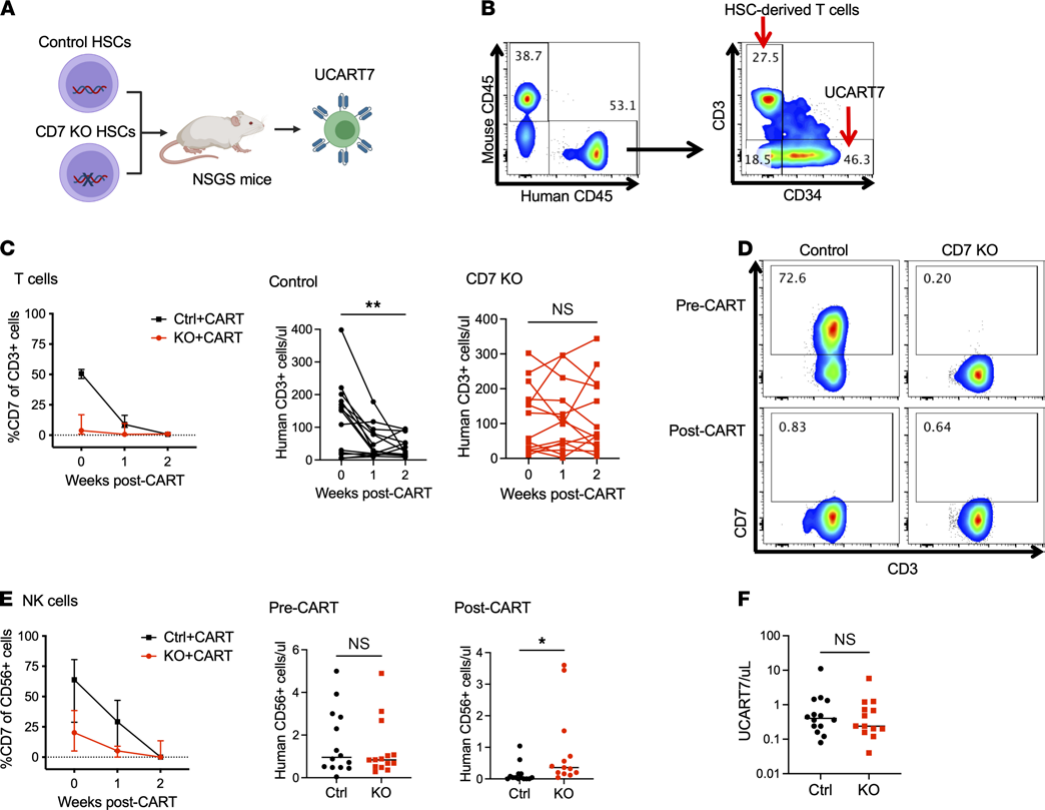
T cells and NK cells are better preserved in mice with CD7-KO HSCs after UCART7 treatment. (**A**) Schema: NSGS mice engrafted with either control or CD7-KO HSCs were treated with UCART7 after 15 weeks of engraftment (*n* = 14/group). (**B**) Gating strategy to identify the different T cell populations. Within the human CD45^+^ cell population, HSC-derived T cells express CD3, while UCART7 lacks CD3 but can be identified by expression of human CD34, a selection marker inserted with the CAR. (**C**) Peripheral blood CD7^+^ expression and numbers of T cells in control and CD7-KO HSC–engrafted mice. (**D**) Representative flow cytometry plots showing CD7 expression in T cells before and after UCART7 treatment. (**E**) CD7 expression on peripheral blood NK cells, and numbers before and after UCART7 treatment. (**F**) Peripheral blood UCART7 numbers 1 week after injection. Statistical analyses were performed using paired 2-tailed Student’s *t* test (**C**) or unpaired 2-tailed Student’s *t* test (**E** and **F**). *P < 0.05, **P < 0.01.

We proceeded to inject control or CD7-KO HSC–engrafted mice with UCART7, to ensure that these cells were indeed resistant to CD7-targeting therapy (Figure 5A). UCART7 was generated from allogeneic T cells that were deleted for *TRAC* and *CD7* genes, followed by insertion of a CD7-targeting CAR. T cells derived from the HSCs were easily distinguishable from the adoptively transferred T cells based on expression of CD3 (unique to HSC-derived T cells) and CD34 (a detection marker for UCART7) (Figure 5B). We observed an immediate loss of human CD7^+^ cells in the peripheral blood of both groups of mice, and this loss led to a significant decrease in human T cells in the controls, while T cells were maintained in mice engrafted with CD7-KO HSCs (Figure 5, C and D). Peripheral blood NK cells were also better maintained in mice with CD7-KO HSCs as compared with mice with control HSCs after UCART7 treatment (Figure 5E). Of note, we detected minimal numbers of circulating UCART7 cells in these experiments, and this was not significantly different between the 2 groups (Figure 5F).

**Figure 6:**
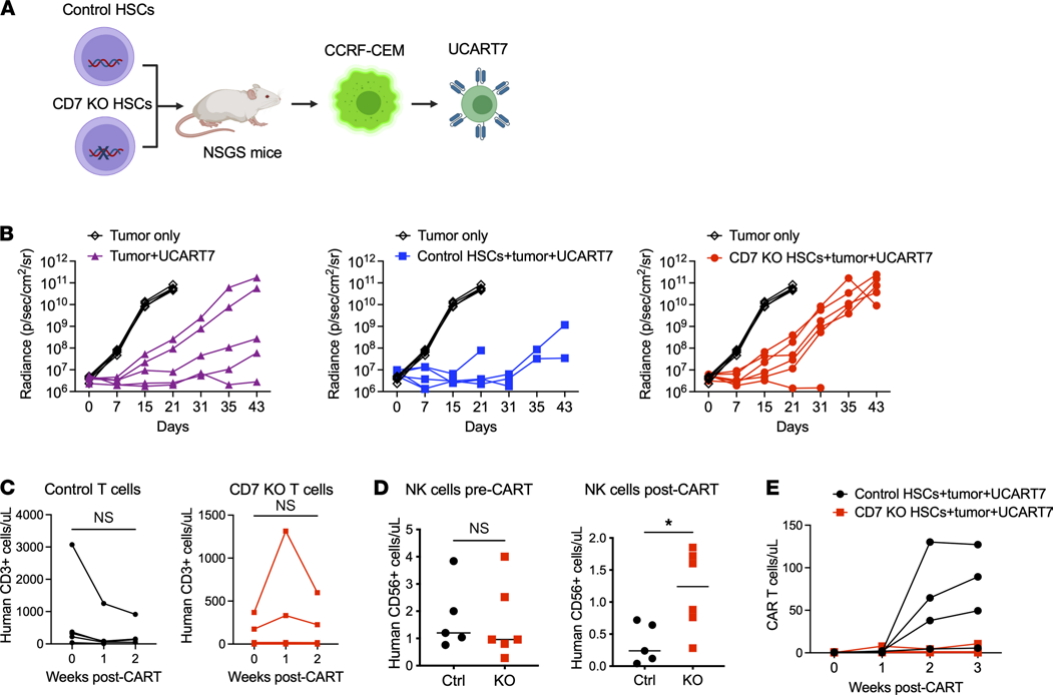
UCART7 antitumor efficacy is not impaired by the presence of CD7-KO hematopoiesis. (**A**) Schema: NSGS mice engrafted with either control or CD7-KO HSCs were injected with CCRF-CEM, a CD7^+^ T-ALL cell line, followed by treatment with UCART7. A separate cohort of mice were injected with CCRF-CEM only, or CCRF-CEM and UCART7 without prior HSC engraftment (*n* = 5–6/group). (**B**) Serial tumor burden measured by bioluminescence imaging for each treatment group, with tumor-only control as reference. Each line represents 1 mouse. (**C**) Peripheral blood T cell counts in control or CD7-KO HSC and tumor-engrafted mice. (**D**) NK cells before and after UCART7 treatment in mice with HSCs and concurrent tumor. (**E**) UCART7 expansion in peripheral blood. Statistical analyses were performed using paired 2-tailed Student’s *t* test (**D**) or unpaired 2-tailed Student’s *t* test (**E**). *P < 0.05.

To ensure that the antitumor activity of UCART7 is preserved in the setting of CD7-KO HSCs, we proceeded to add CCRF-CEM, a CD7^+^ T-ALL cell line, to the HSC-engrafted mice prior to UCART7 injection (Figure 6A). Tumor control was comparable in mice with control or CD7-KO HSCs as compared with a cohort of mice without prior HSC engraftment (Figure 6B). We observed a trend toward reduction of T cell numbers in the control HSC-engrafted mice, although this was not statistically significant, while T cell numbers were stable in CD7-KO HSC–engrafted mice (Figure 6C). Control HSC-engrafted mice had the expected loss of NK cells, while in CD7-KO HSC–engrafted mice, NK cells could still be detected (Figure 6D). Intriguingly, UCART7 expansion was more robust in mice with control HSCs than with CD7-KO HSCs (Figure 6E).

### Targeted deep sequencing does not reveal off-target mutations in CD7-KO HSCs.

Off-target mutations is a concern when performing gene editing in HSCs, as deleterious mutations in HSCs can have long-term and profound consequences. We previously performed GUIDE-seq (16), an unbiased genome-wide method to identify DNA double-strand breaks, in primary human T cells to identify off-target mutations after CRISPR/Cas9 editing of *CD7*. We interrogated the 4 off-target mutation sites identified by GUIDE-seq in CD7-KO HSCs, and we also added 5 off-target sites that were predicted in silico to have a high degree of homology to the CD7 gRNA. NGS across these 9 locations in 3 different primary human CD34^+^ cells after *CD7* editing revealed no off-target mutations, despite a high degree of on-target mutations (Table 1).

## Discussion

CD7 is an attractive target for immunotherapy of T cell malignancies, as it is broadly expressed across a wide range of T cell lineage tumors. However, CD7 is also present on the majority of normal T cells and NK cells, and targeting CD7 could lead to prolonged immunosuppression. Our work examines whether treatment with CD7-targeting CAR T cells could lead to intolerable toxicity and, if so, whether we could circumvent this by deleting the *CD7* gene from HSCs to generate T and NK cells lacking CD7 expression but still capable of host protection.

We first examined whether the CD7^–^ T cells that are found in healthy subjects could be sufficient to provide protective immunity after CD7-targeting CAR T cell treatment. In healthy adults, elimination of all CD7^+^ cells will lead to complete loss of NK cells and loss of 90% of T cells. The remaining CD7^–^ T cells predominantly show the CD4^+^ effector memory phenotype, and while they retain effector functions such as cytokine production and cytotoxicity, they do not expand robustly in response to CD3/CD28 stimulation. Our results corroborate prior studies of CD7^–^ T cells that reported increased expression of CD4 and CD45RO and a lower proliferative response to various mitogens (8, 17). Furthermore, we observed that 30%–50% of CD7^–^ T cells can express CD7 and become susceptible to CD7-targeting CAR T cells after activation, leading to further decrease of the functional T cell repertoire.

Of note, CD7^–^ T cells are not present in cord blood, and single-cell RNA-seq data of normal PBMCs show lower levels of CD7 transcript expression in T cells from older individuals. Supercentenarians, defined as individuals of age greater than 110, have particularly low levels of CD7^+^ T cells. We can hypothesize from these findings that loss of CD7 may be a surrogate marker for aging of the T cell repertoire. Practically, our findings suggest that CD7-targeting therapy may lead to more profound T cell depletion in pediatric patients, and these patients may be in the most need for strategies to circumvent T cell toxicity.

Therefore, we proceeded to generate CD7-KO HSCs, with the goal of creating CD7-KO T and NK cells that were resistant to CD7 targeting therapy while still retaining function. We examined the engraftment and differentiation profile of CD7-KO HSCs in 3 different strains of mice — NSG, NSGS, and NSG–huIL-15 — to ensure that these cells retained their ability to reconstitute the full spectrum of human hematopoiesis. The hematopoietic system arising from CD7-KO HSCs was nearly identical to that of control HSCs, regardless of host mouse strain, with the exception of decreased surface CD7 expression in T and NK cells derived from CD7-KO HSCs. While the extent of CD7 mutations detected in each mouse diverged over time, likely representing outgrowth of dominant clones from the initial HSC product, the average mutation rate within each experiment remained stable. Therefore, our results at this time do not support any fitness advantage or disadvantage of *CD7* mutations on the hematopoietic system.

Human T cells derived from CD7-KO HSCs did not exhibit any functional deficiencies in terms of intracellular signaling, degranulation, cytokine production, and response to IL-7 stimulation. CD7-KO NK cells were also comparable with control NK cells in terms of cytotoxicity, surface marker expression, and IL-7 response. One point that was particularly interesting to us was that the properties of CD7^–^ T cells, such as lack of CCR7 expression and reduced proliferation, were not recapitulated in CD7-KO T cells. These results indicate that CD7-KO T cells more closely resemble bulk T cells (that are predominantly CD7^+^) than physiologically CD7^–^ T cells, and they provide evidence that CD7-KO T cells could restore immune function after CD7-targeting therapy that would otherwise be limited to the function of CD7^–^ T cells alone. Of note, multiple groups have independently generated methods to remove CD7 from mature T cells so that they could be generated into CD7-targeting CAR T cells, and they have shown that both the proliferative and cytotoxic functions of CD7-KO CAR T cells remain intact (4–6).

One limitation of our studies is that the T cell engraftment was highly variable, ranging from 0.6% to 97% of human cells in the peripheral blood in NSGS mice. This may be because the ex vivo gene editing prior to injection decreased the repopulating capacity of the cord blood CD34^+^ cells, and higher cell doses may be necessary to improve the reliability of T cell engraftment. Several of our experiments required large numbers of mice to meet statistical significance due to this variability within the experimental model. Therefore, we cannot exclude the possibility that subtle qualities unique to CD7-KO T cells may exist, but these are buried within the variability of the system.

An additional limitation is that the majority of our experiments were performed in NSGS mice, as this strain supports rapid and robust T cell engraftment but only expresses CD7 in around 50% of the human T cells that emerge after HSC engraftment. This is likely due to high proportions of CD4^+^ and CD45RO^+^ T cells in these mice (18), 2 markers that inversely correlate with CD7 expression. In this setting, it is difficult to ascertain the function of CD7-KO T cells solely based on the absence of CD7 expression, since this will be a mixed population that contains WT CD7^–^ T cells and CD7-KO T cells; therefore, again, our studies would not be able to detect subtle differences present only in CD7-KO T cells. Additionally, T cells generated in humanized NSGS mice are not fully functional due to lack of thymic education, precluding our ability to perform in vivo functional experiments, such as immunization or skin grafts. Further investigations with more advanced models will be required to address these limitations.

Therefore, we do not preclude the possibility that lack of CD7 may still have functional consequences that may perhaps manifest when T or NK cells are challenged with specific biologic stressors. On this note, CD7 is known to contribute to T and NK cell activation in vitro (19, 20), and while genetic deletion of CD7 in mice does not impair T cell development or major functions (21, 22), CD7-KO mice have been shown to be resistant to LPS-induced shock (23). In humans, the full impact of CD7 deletion may well only manifest after long-term follow-up of patients after CD7-KO HSC transplantation. The risks of this treatment will need to be balanced carefully with the potential benefits for patients with incurable T cell malignancies.

The addition of UCART7 to HSC-engrafted mice led to reduction of T cells and NK cells in mice with control HSCs, while these cells were better preserved in mice with CD7-KO HSCs. In the non–tumor-bearing mice, these results were statistically significant, despite the variability of T cell engraftment and the high percentage of CD7^–^ T cells in mice with control HSCs. Injection of CCRF-CEM tumor cells prior to UCART7 treatment still led to the expected decrease in human T and NK cells without any compromise of antitumor effect. It is intriguing that mice with CD7-KO HSCs and tumor, followed by UCART7 treatment, had less detectable UCART7 expansion in the peripheral blood than mice with control HSCs. This may be due to decreased antigen burden seen by UCART7 when CD7 is deleted, or alternatively due to rejection of UCART7 by the UCART7-resistant CD7-KO T and NK cells. We hope to investigate the mechanism behind these findings in future studies.

Finally, we note that gene editing of HSCs is still in early stages, and there are understandable trepidations when applying this modality in human subjects. In particular, CRISPR-Cas9 gene editing of HSCs has only recently entered human clinical trials (24, 25), and we still await the long-term effects of this technology. Investigations to date have shown that CRISPR-Cas9 gene editing can be done in HSCs without undue toxicity from the editing procedure. However, off-target genotoxicity remains a major concern, as this will be specific to the gRNA used in each experiment and cannot be broadly generalized as safe. On this note, we looked at several off-target sites related to the CD7 gRNA that were identified both experimentally through GUIDE-seq in primary human T cells and computationally through in silico prediction models. We did not find any off-target editing primary human CD34^+^ cells in any of the predicted off-target sites that we queried, despite high levels of on-target mutations. These results are consistent with other studies investigating CRISPR/Cas9 off-target activity in HSCs (25–27), where minimal off-target events were detected as long as the gRNA was chosen carefully. However, this does not preclude the possibility of off-target sites unique to HSCs that were not identified through our prediction models, or of off-target editing specific to individuals with certain single-nucleotide variations. All of these possibilities will need to be kept in mind when utilizing this technology.

In summary, we show that CD7-KO HSCs can generate functional T cells that lack CD7 expression and are, thus, resistant to CD7-targeting CART treatment. These results advance the concept of genetic deletion of cell surface markers in HSCs as a strategy to avoid the hematopoietic toxicity of antigen-specific immunotherapy. Prior work in this field has focused solely on CD33, a myeloid antigen, confining the reach of this strategy to myeloid malignancies. We now show that CD7, a T cell antigen, can also be a viable target, opening up the field to T cell malignancies. We can further postulate that, potentially, a large number of cell-surface antigens exist that may be safely removed from HSCs without untoward effects, and this may be exploited therapeutically in the future, especially as we gain more clinical experience with gene-edited HSCs.

## Methods

### Evaluation of CD7^–^ T cells.

Normal donor PBMCs were obtained from leukoreduction chambers of anonymous platelet donors. Cord blood units were obtained from the St. Louis Cord Blood Bank (St. Louis, Missouri, USA). To separate CD7^+^ and CD7^–^ T cell fractions, T cells were first purified from PBMCs using a Human Pan T Isolation Kit (Miltenyi Biotec, 130-096-535) and separated on AutoMACS Pro Separator (Miltenyi Biotec). T cells were then labeled with CD7-biotin, followed by Anti-Biotin Microbeads (Miltenyi Biotec, 130-090-485), and positive and negative fractions were collected from the AutoMACS. Unselected T cells, CD7^+^ , and CD7^–^ T cells were incubated with anti-CD3/CD28 beads (Thermo Fisher Scientific, 40203D) at a 3:1 bead/cell ratio and cultured in RPMI-1640 medium (Corning, 10-040-CM) containing 10% FBS (Thermo Fisher Scientific, 10-082-139), 1% penicillin/streptomycin (Corning, 30-002-CI), 1% L-glutamine (Invitrogen, 35050079), 1% sodium pyruvate (Corning, 25-000-CI), 1% MEM nonessential amino acids (Corning, 25-050-CI), 2% HEPES buffer (Corning, 25-060-CI), and 50 µM 2-mercaptoethanol (Thermo Fisher Scientific, 21985-023). Cells were counted every 2–3 days, and CD7 expression was assessed serially by flow cytometry. For CAR T cell generation, bulk T cells, CD7^+^ and CD7^–^ T cells were transduced with lentivirus containing previously published CAR19 (28) or CAR33 (29) constructs 1 day following activation.

### Single-cell RNA-seq data from PBMCs.

Single-cell RNA-seq data of healthy donor PBMCs were downloaded from NCBI Gene Expression Omnibus (accession nos. GSE171555, GSE166489) and from http://gerg.gsc.riken.jp/SC2018/, and they were imported into Partek Flow Genomic Analysis Software. Raw count matrix files were filtered to only include cells with < 20% human mitochondrial DNA content and with a minimum of 200 and maximum of 20,000 expressed genes. Genes were filtered to only include those with expression levels > 1 in at least 99.9% of cells, followed by normalization. T cells within each sample were identified by expression of *TRAC* and *CD3E* genes and were further classified into CD4^+^ and CD8^+^ T cells by expression of *CD4* and *CD8A*. Cells with *CD7* gene expression were quantified within the CD4^+^ and CD8^+^ T cell populations. Differential gene expression was performed using Partek gene-specific analysis (GSA) comparing CD7^+^ and CD7^–^ T cells within the 3 adult healthy donors in the GSE166489 data set, which was selected due to concurrent surface protein and gene expression evaluation.

### Generation of UCART7.

UCART7 was manufactured as previously described (4), with the following modifications. CD4^+^ and CD8^+^ T cells were selected from normal donor PBMCs and mixed at a 1:1 ratio, and they were activated with Transact (20 μL/mL, Miltenyi Biotec), followed by culture in Texmacs media supplemented with 10 ng/mL IL-7 and 10 ng/mL IL-15. Two days after activation, T cells were washed and electroporated in 100 μL Maxcyte EP buffer containing 15 μg TrueCut Cas9 Protein v2 (Thermo Fisher Scientific, A36499) and 20 μg of each gRNA targeting *CD7* and *TRAC* (Trilink) using the Maxcyte GT expanded T cell program #2. Two hours following electroporation, cells were transduced with a lentiviral vector containing a second-generation CD7-CAR with a 4-1BB cosignaling domain, which also contained the extracellular domain of human CD34 via a P2A peptide to enable detection and purification of CAR^+^ cells. CAR T cells were expanded for 10–14 days prior to i.v. injection into mice (2 *×* 10^6^ CAR^+^ cells/mouse) previously engrafted with control or CD7-KO HSCs. Residual CD3^+^ cells after gene editing were depleted from the UCART7 product using Human CD3 Microbeads (Miltenyi Biotec, 130-050-101).

### UCART7 in vitro cytotoxicity.

Unselected T cells, CD7^+^ or CD7^–^ T cells from 4 different donors that had previously been expanded and frozen were thawed and incubated in vitro either alone or with UCART7 at a 1:1 E:T ratio for 48 hours in triplicate. Cells were then harvested and stained for flow cytometry using the following antibodies: CD3-PE/Cy7 (clone UCHT1, eBioscience, 25-0038-41), CD7-APC (clone M-T701, BD Biosciences, 561604), CD34-PE (clone QBEnd10 + Immu133 + Immu409, Beckman Coulter, IM1459U), and LIVE/DEAD fixable yellow (Thermo Fisher Scientific, L34967). Percent reduction of T cells were calculated by dividing the number of CD3^+^ T cells remaining in each group after UCART7 treatment over the number of CD3^+^ T cells remaining in each group without UCART7 treatment.

### Gene editing of HSCs.

On day 1, human CD34^+^ cells were purified from fresh cord blood units using a CD34 MicroBead Kit (Miltenyi Biotec, 130-046-702). Cells were cultured overnight in StemSpan SFEM (Stemcell Technologies, 09600) with 100 ng/mL of stem cell factor (SCF; Peprotech, 300-07), Flt3-ligand (Peprotech, 300-19), and thrombopoietin (Peprotech, 300-18). On day 2, CD34^+^ cells were nucleofected per manufacturer’s instructions with the P3 Primary Cell Nucleofection Kit (Lonza, V4XP-3012), with the addition of 25 μg of TrueCut Cas9 Protein v2 (Thermo Fisher Scientific, A36499) and 50 μg of CD7-gRNA (custom synthesized from Trilink), using a 4D-Nucleofector Core Unit (Lonza, AAF-1002B) program EO-100. The CD7-gRNA sequence is as follows: 5′-ATCACGGAGGTCAATGTCTA-3′. Of note, 2′-O-methyl and 3′ phosphorothioate bases were incorporated at the 3 terminal bases of the 5′ and 3′ ends of the gRNA to increase stability. Control CD34^+^ cells were nucleofected with Cas9 protein only. Cells were incubated at 32°C overnight. On day 3, cells were injected i.v. into mice as detailed below. A small fraction of cells was plated on Human Methylcellulose Enriched Media (R&D Systems, HSC005), and colonies were harvested after 14–16 days. DNA was extracted for evaluation of *CD7* gene mutations by NGS.

### Molecular analysis.

Genomic DNA was isolated from colonies derived from HSCs in vitro*,* or peripheral blood obtained from mice engrafted with HSCs in vivo, using the DNeasy Blood & Tissue Kit (Qiagen, 69504). Samples were submitted to the Washington University Genome Engineering and iPSC Center (St. Louis, Missouri, USA) for NGS to detect mutations in the *CD7* gene.

### In vivo engraftment of HSCs.

Nonobese diabetic (NOD)/severe combined immunodeficient (SCID)/common γ chain^–/–^(γc^−/−^) (NSG, The Jackson Laboratory, 005557), NSG–huIL-15 (NSG mice expressing human IL-15; The Jackson Laboratory, 030890), or NSGS mice (NSG mice expressing human IL-3, GM-CSF, and SCF; The Jackson Laboratory, 013062) were sublethally irradiated with 250 cGy followed by i.v. injection with 5 *×* 10^4^ to 1 *×* 10^5^ CD34^+^ cells per mouse. Human cell engraftment was assessed by serial blood collections at 4, 8, 12, 15–16, and 19–20 weeks after CD34^+^ cell injection.

### T cell in vitro functional studies.

Splenocytes harvested from control or CD7-KO HSC–engrafted mice were used for the following studies. Detection of intracellular phosphorylated proteins (pErk1/2, pSTAT5) in response to PMA (400 nM, Sigma-Aldrich, P8139-1MG) or IL-2 (100 ng/mL, PeproTech, 200-02-1 mg) was performed using the BD Phosflow T Cell Activation Kit (BD Biosciences, 560750) according to the manufacturer’s instructions. Measurement of degranulation and intracellular cytokine production was performed by treating cells with PMA (50 nM) and ionomycin (1 μg/mL) and incubating with CD107a-FITC (clone eBioH4A3, eBioscience, 11-1079-41), monensin, anti-CD49d (clone 9F10, BD Biosciences, 555501), and anti-CD28 (clone CD28.2, BD Biosciences, 555725) for 4 hours. Cells were then harvested and stained with CD3-PE/Cy7 (clone UCHT1, eBioscience, 25-0038-41), CD7-APC (clone M-T701, BD Biosciences, 561604), and LIVE/DEAD fixable yellow (Thermo Fisher Scientific, L34967). Subsequently, cells were treated with the Invitrogen Fix & Perm Cell Permeabilization Kit (Thermo Fisher Scientific, GAS003), followed by intracellular staining with IFN-γ–PE (clone 4S.B3, BioLegend, 502510), GM-CSF–BV421 (clone BVD2 21C11, BD Biosciences, 562930), TNF-α–AF700 (clone Mab11, BioLegend, 502928), MIP1b-APC/H7 (BD Biosciences, 561280), and IL-2–PE–CF594 (clone 5344.111, BD Biosciences, 562384). Samples were analyzed by flow cytometry after staining completed.

### Expansion of CD7-KO T cells.

Human T were sorted from splenocytes harvested from control or CD7-KO HSC–engrafted NSGS mice based on expression of human CD45 and CD3, using a MoFlo cell sorter (Beckman Coulter) or a SY3200 Synergy HAPS cell sorter (Sony Biotechnology). Cells were cultured in R10 media with the addition of anti-CD3/CD28 beads at a 3:1 bead/cell ratio, and they were counted every 2 days to maintain cell density less than 1 *×* 10^6^ cells/mL. Flow cytometry was performed 3 days after activation to assess CD7 expression.

### NK cell in vitro tumor killing assay.

Human NK cells were sorted from splenocytes harvested from control or CD7-KO HSC–engrafted NSG-huIL-15 mice based on expression of human CD45 and CD56, using a SY3200 Synergy HAPS cell sorter (Sony Biotechnology). Control and CD7-KO NK cells were evaluated using IncuCyte Live-cell Analysis system. In total, 5000 UM-SCC-9 cells expressing CBR/GFP were incubated for 2 hours in a 96-well plate and imaged prior to the addition of the NK cells at a 5:1 and 10:1 E:T ratio. Real-time images were captured every 3 hours and up to 72 hours, and they were analyzed using the Incucyte software. Data are presented as green object counts (GFP^+^ UM-SCC-9 tumor cells).

### Administration of NT-I7 to HSC-engrafted mice.

NSGS mice were engrafted with control or CD7-KO HSCs followed by serial injections of 10 mg/kg NT-I7 (NeoImmuneTech Inc.) s.c. starting 1 week after CD34^+^ cell injection. NT-I7 injections were continued every 2 weeks for 7 doses, total. Blood was collected from the mice at 4, 8, 12, and 15 weeks after CD34^+^ cell injection as detailed above.

### In vivo treatment with UCART7.

T cell engraftment was confirmed in NSGS mice at approximately 15–16 weeks after CD34^+^ cell injection, after which, 2 *×* 10^6^ UCART7 cells were injected i.v. and blood was collected weekly for 2–4 weeks. Alternatively, mice were injected with 2 *×* 10^5^ CCRF-CEM cells (ATCC, CCL-119) that had been engineered to express CBR/GFP to facilitate tracking of tumor. UCART7 was injected 1 week after tumor was established. Tumor burden was monitored longitudinally by bioluminescence imaging using an Ami HT optical imaging system (Spectral Instruments).

### Flow cytometry.

The following antibodies were used to assess %CD7 on PBMCs by flow cytometry: CD3-PE/Cy7 (clone UCHT1, eBioscience, 25-0038-41), CD4-FITC (clone RPA-T4, BioLegend, 300506), CD7-APC (clone M-T701, BD Biosciences, 561604), CD8-BV421 (clone RPA-T8, BioLegend, 301036), CD56-PE (clone 5.1H11, BioLegend, 362508), and LIVE/DEAD fixable yellow (Thermo Fisher Scientific, L34967).

Memory subsets on T cells were quantified using the following antibodies: CD3-PE/Cy7 (clone UCHT1, eBioscience, 25-0038-41), CD4-BV650 (clone SK3, BD Horizon, 563875), CD7-PE (clone M-T701, BD Biosciences, 555361), CD8-AF700 (clone HIT8a, BioLegend, 300920), CD56-FITC (clone HCD56, BioLegend, 318304), CD45RO-BV421 (clone UCHL1, BioLegend, 304224), CD197-AF647 (clone G043H7, BioLegend, 353218), and LIVE/DEAD fixable yellow (Thermo Fisher Scientific, L34967).

Peripheral blood obtained from mice were lysed in ACK lysis buffer, and human cell engraftment was assessed by flow cytometry using the following antibodies: CD3-PE/Cy7 (clone UCHT1, eBioscience, 25-0038-41), CD4-FITC (clone RPA-T4, BioLegend, 300506), CD7-APC (clone M-T701, BD Biosciences, 561604), CD8-PerCP/Cy5.5 (clone RPA-T8, BioLegend, 301032), CD19-BV605 (clone HIB19, BioLegend, 302244), CD33-BV711 (clone WM53, BD Biosciences, 563171), mouse CD45-APC/Cy7 (clone 30-F11, BioLegend, 103116), human CD45-BV421 (clone HI30, BioLegend, 304032), CD56-PE (clone 5.1H11, BioLegend, 362508), and LIVE/DEAD fixable yellow (Thermo Fisher Scientific, L34967).

After UCART7 injections, the panel was modified as follows: CD3-PE/Cy7 (clone UCHT1, eBioscience, 25-0038-41), CD4-BV650 (clone SK3, BD Horizon, 563875), CD7-APC (clone M-T701, BD Biosciences, 561604), CD34-PE (clone QBEnd10 + Immu133 + Immu409, Beckman Coulter, IM1459U), CD56-FITC (clone HCD56, BioLegend, 318304), mouse CD45-APC/Cy7 (clone 30-F11, BioLegend, 103116), human CD45-BV421 (clone HI30, BioLegend, 304032), and LIVE/DEAD fixable yellow (Thermo Fisher Scientific, L34967).

Mice injected with tumor followed by UCART7 were assessed with the following panel: tumor-GFP, CD3-PE/Cy7 (clone UCHT1, eBioscience, 25-0038-41), CD4-PerCP/Cy5.5 (clone RPA-T4, BioLegend, 300530), CD7-APC (clone M-T701, BD Biosciences, 561604), CD34-PE (clone QBEnd10 + Immu133 + Immu409, Beckman Coulter, IM1459U), CD56-BV605 (clone HCD56, BioLegend, 318334), mouse CD45-APC/Cy7 (clone 30-F11, BioLegend, 103116), human CD45-BV421 (clone HI30, BioLegend, 304032), and LIVE/DEAD fixable yellow (Thermo Fisher Scientific, L34967).

Human NK cells derived from control or CD7-KO HSCs were assessed using the following antibodies: CD16-FITC (clone 3G8, Thermo Fisher Scientific, MHCD1601), CD3-ECD (clone UCHT1, Beckman Coulter, IM2705U), mouse CD45-PerCP/Cy 5.5 (clone 30-F11, BD Biosciences, 550994), CD56-PE/Cy7 (clone N901, Beckman Coulter, A51078), CD7-APC (clone M-T701, BD Biosciences, 561604), KIR2DL1/DS1-VioBlue (clone 11PB6, Miltenyi, 130-125-246), KIR2DL2/L3/S2-BV421 (clone CH-L, BD Pharmingen, 743451) KIR3DL1-BV421 (clone DX9, BD Pharmingen, 742979), human CD45-KO (clone J33, Beckman Coulter, A96416), and LIVE/DEAD zombie NIR (BioLegend, 423105).

Samples were run on an Attune NxT Flow Cytometer (Thermo Fisher Scientific) or a Gallios Flow Cytometer (Beckman Coulter). Analysis was performed using FlowJo 10.6.1 (Tree Star Inc.).

### Off-target mutation analysis.

In silico off-target sites (Table 1) were selected using 2 different prediction algorithms: CCTop (https://cctop.cos.uni-heidelberg.de:8043/) (30) and Cas-OFFinder (http://www.rgenome.net/cas-offinder/) (31). GUIDE-seq sites were previously published. A full list of off-target sequences queried are as follows: CD7, ATCACGGAGGTCAATGTCTA (on-target); PACRG, TTTTATGGTTATCTTTATAA (GUIDEseq); ARHGEF4, CAAACGATATTGTGGCCAAA (GUIDEseq); HMGB1P22, TTTAAAATAATTAATAATAC (GUIDEseq); CCBE1, TCCCGGTCATATCGGTATCC (GUIDEseq); AC016907.3, CACAAGGAAGTCAATGTCTA (in silico); RP11-92A5.2, ATCAGGGAGGAAAATGTCTA (in silico); CTA-254O6.1, AGGACAGAGATCAATGTCTA (in silico); RPS3AP52, ATCTAGAGGGTCAATGTCTA (in silico); and FUNDC2P2, ATCACGGAGGGACAATTTCTA (in silico).

Genomic DNA was obtained from matched donor control or CD7-KO HSCs after in vitro differentiation on methylcellulose. NGS was performed after PCR amplification of each off-target site. Read counts at each off-target site ranged from 173 to 9911 (median, 6185). When assessing for mutations, we excluded single nucleotide polymorphisms that were defined as single-bp changes occurring in both control and KO samples for an individual donor at approximately 50% or 100% frequency.

### Statistics.

All statistical analyses were performed using GraphPad Prism v9 for macOS. Unpaired, 2-tailed Student’s *t* test, paired 2-tailed Student’s *t* test, 1-way ANOVA, and 2-way ANOVA were used to compare groups as detailed in the figure legends. Significance was defined as a *P* value of less than 0.05.

### Study approval.

All animal studies were approved by the IACUC at Washington University (protocol no. 20180005).

## Author contributions

MYK contributed conceptualization, methodology, formal analysis, investigation, data curation, writing of the original draft, and review and editing of the manuscript. MLC contributed conceptualization, methodology, investigation, resources, and review and editing of the manuscript. MTJ contributed investigation, and review and editing of the manuscript. JKR contributed investigation, and review and editing of the manuscript. JH contributed investigation. TAF contributed investigation, and review and editing of the manuscript. JFD contributed review and editing of the manuscript, supervision, and funding acquisition.

## Supplementary Material

Supplemental data

## Figures and Tables

**Table 1:**
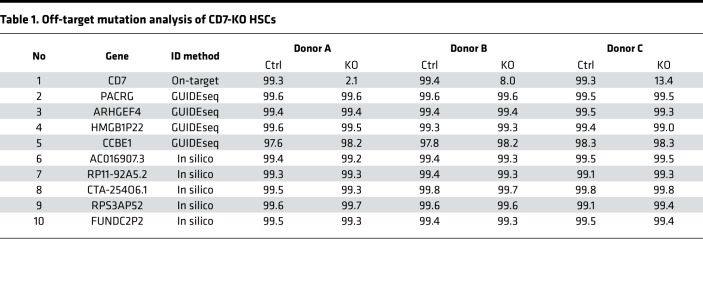
Off-target mutation analysis of CD7-KO HSCs

## Version 1 08/23/2021

Electronic publication
