# Supplementary material for: CD7-deleted hematopoietic stem cells can restore immunity after CAR T cell therapy
Source: JCI Insight. 2021 Aug 23;6(16):e149819. doi: 10.1172/jci.insight.149819 (PMC8410010; doi:10.1172/jci.insight.149819)
Supplement: Supplemental data [file jciinsight-6-149819-s176.pdf]

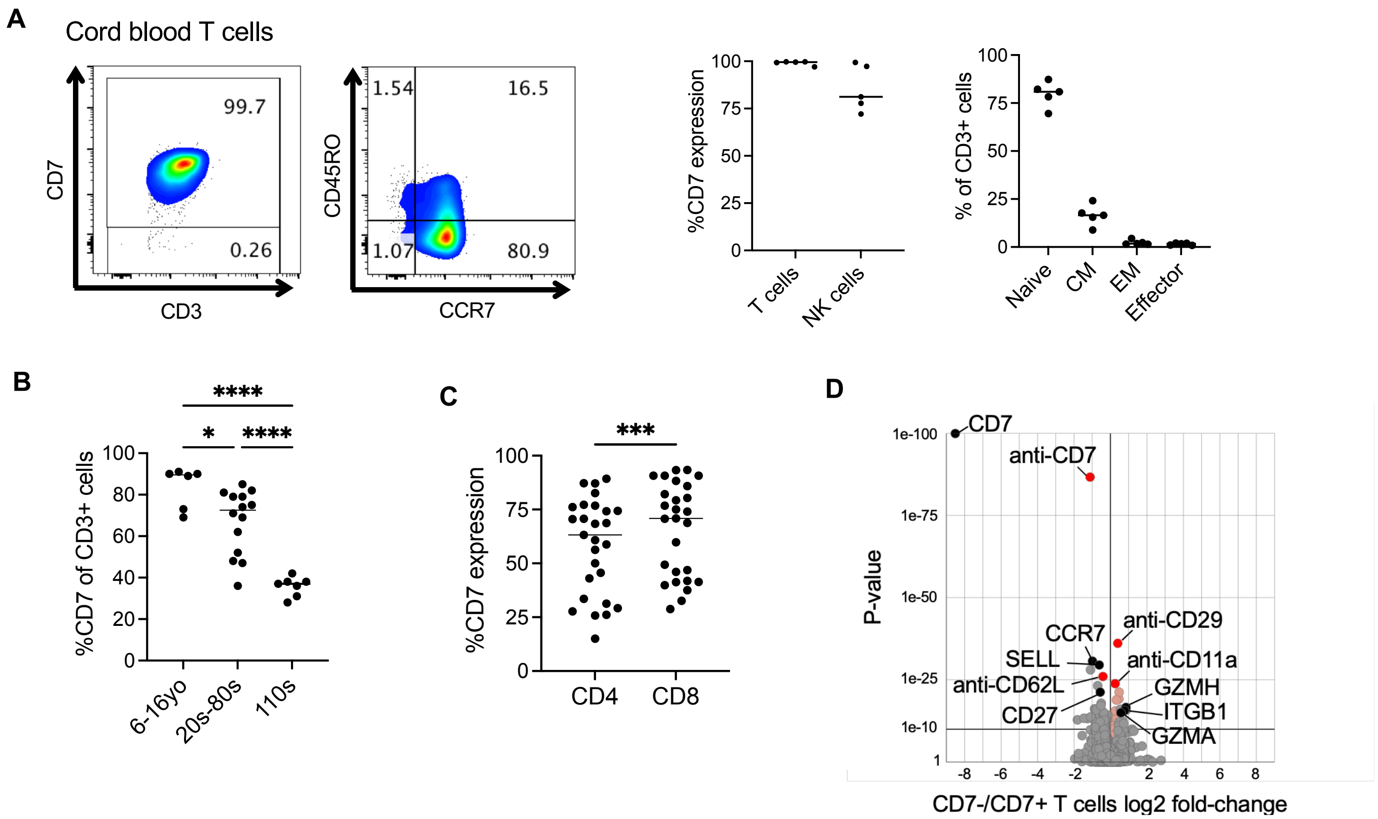

Supplemental Figure 1. CD7 expression in T cells decreases with age. (A) Cord blood T cells uniformly express CD7 and have a predominantly naïve phenotype (n=5). (B) CD7 transcript levels in T cells were quantified in single-cell RNA-seq datasets of healthy donor PBMCs from various age groups (6-16yo: n=6, 20s-80s: n=14, 110s: n=7). (C) CD7 transcript levels are higher in CD8+ T cells than CD4+ T cells. (D) Volcano plot comparing surface protein expression and gene expression profile of CD7-negative T cells to CD7+ T cells from CITE-seq data of healthy PBMCs (n=3). P-values were determined by one-way ANOVA with Tukey's multiple comparisons test (B) or paired Student's *t*-test (C). ns=non-significant, *p<0.05, **p<0.01, ***p<0.001, ****p<0.0001.

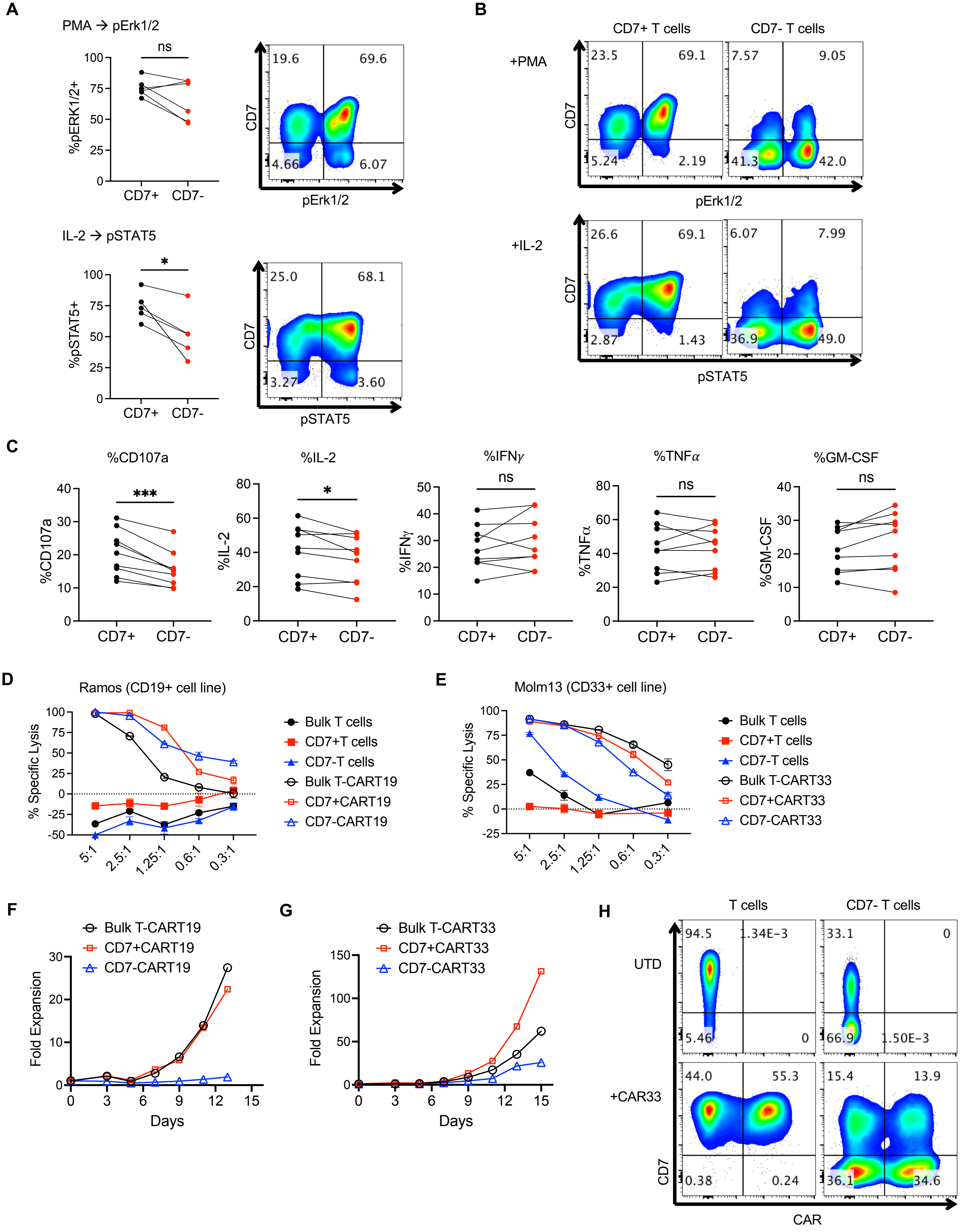
A
PMA → pErk1/2
ns
%pERK1/2+
CD7+
CD7-
19.6
69.6
4.66
6.07
CD7
pErk1/2
IL-2 → pSTAT5
*
%pSTAT5+
25.0
68.1
3.27
3.60
pSTAT5
B
CD7+ T cells
CD7- T cells
+PMA
23.5
69.1
5.24
2.19
7.57
9.05
41.3
42.0
+IL-2
26.6
69.1
2.87
1.43
6.07
7.99
36.9
49.0
C
%CD107a
***
%IL-2
*
%IFNγ
ns
%TNFα
ns
%GM-CSF
ns
D
Ramos (CD19+ cell line)
% Specific Lysis
5:1
2.5:1
1.25:1
0.6:1
0.3:1
Bulk T cells
CD7+T cells
CD7-T cells
Bulk T-CART19
CD7+CART19
CD7-CART19
E
Molm13 (CD33+ cell line)
Bulk T-CART33
CD7+CART33
CD7-CART33
F
Fold Expansion
Days
G
H
T cells
CD7- T cells
UTD
94.5
1.34E-3
5.46
0
33.1
0
66.9
1.50E-3
+CAR33
44.0
55.3
0.38
0.24
15.4
13.9
36.1
34.6
CAR

Supplemental Figure 2. CD7-negative T cells are competent in effector functions. (A) T cells in the CD7-negative fraction can phosphorylate signaling proteins after PMA or IL-2 stimuli (n=6 donors). (B) CD7-negative T cells are able to phosphorylate signaling proteins even after separation from the CD7+ fraction (representative plots from three independent experiments). (C) T cells in the CD7-negative fraction retain degranulation (%CD107a) and cytokine production (IL-2, IFNg, TNFa and GM-CSF) capabilities after PMA/ionomycin stimulation. (D) CD19-targeting CAR T cells (CART19) generated from bulk T cells, CD7+ or CD7- T cells have cytotoxic activity against Ramos, a CD19+ cell line. (E) CD33-targeting CAR T cells (CART33) generated from bulk T cells, CD7+ or CD7- T cells have cytotoxic activity against Molm13, a CD33+ cell line. (F) CART19 generated from CD7-T cells do not proliferate robustly during expansion. (G) CART33 generated from CD7-T cells do not proliferate robustly during expansion. (H) CAR T cells derived from CD7-T cells upregulate CD7 expression after activation. P-values were determined by paired Student's *t*-test. ns=non-significant, *$p<0.05$, **$p<0.01$, ***$p<0.001$.

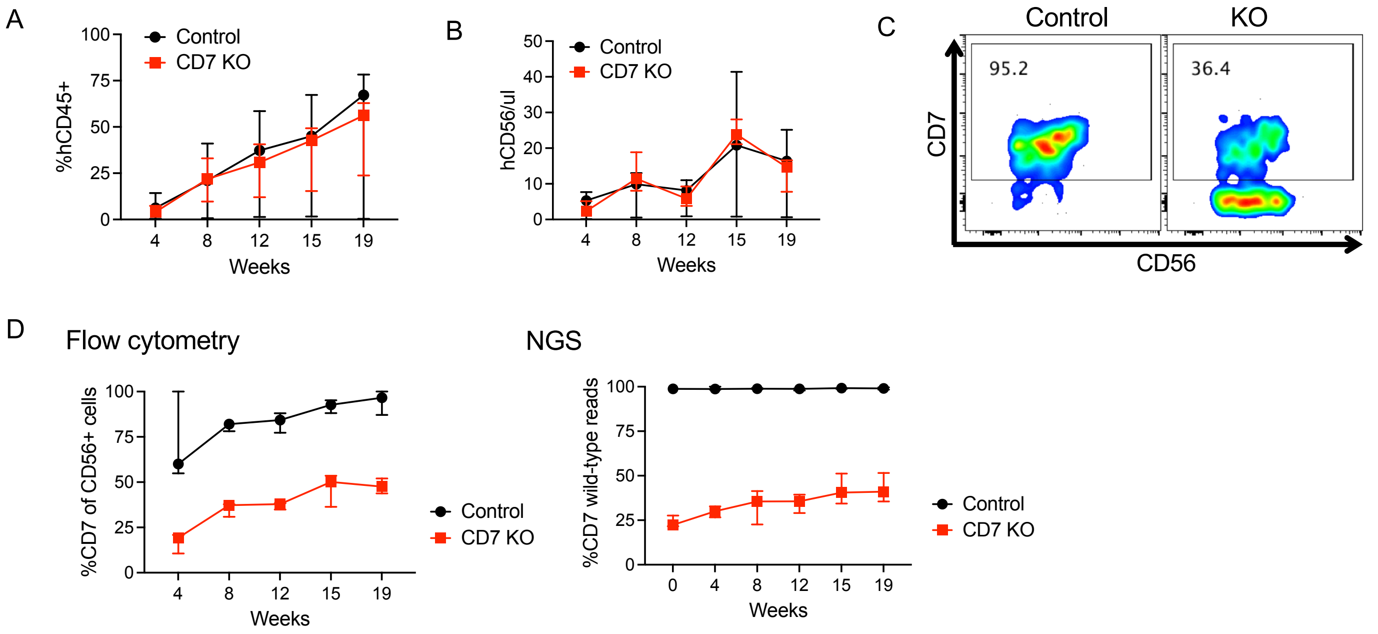

Supplemental Figure 3. CD7 KO HSCs can engraft in NSG-huIL15 mice and generate CD7 KO NK cells. Control or CD7 KO HSCs were injected into NSG-huIL15 mice and engraftment was followed over time (n=5/group). (A) Peripheral blood human CD45+ cells were found at similar frequencies at each timepoint in each group. (B) Levels of human CD56+ NK cells in both groups were similar. (C) Representative flow cytometry plots showing CD7 expression on human NK cells. (D) CD7 expression on NK cells measured by flow cytometry correlates with level of gene mutations detected by NGS.

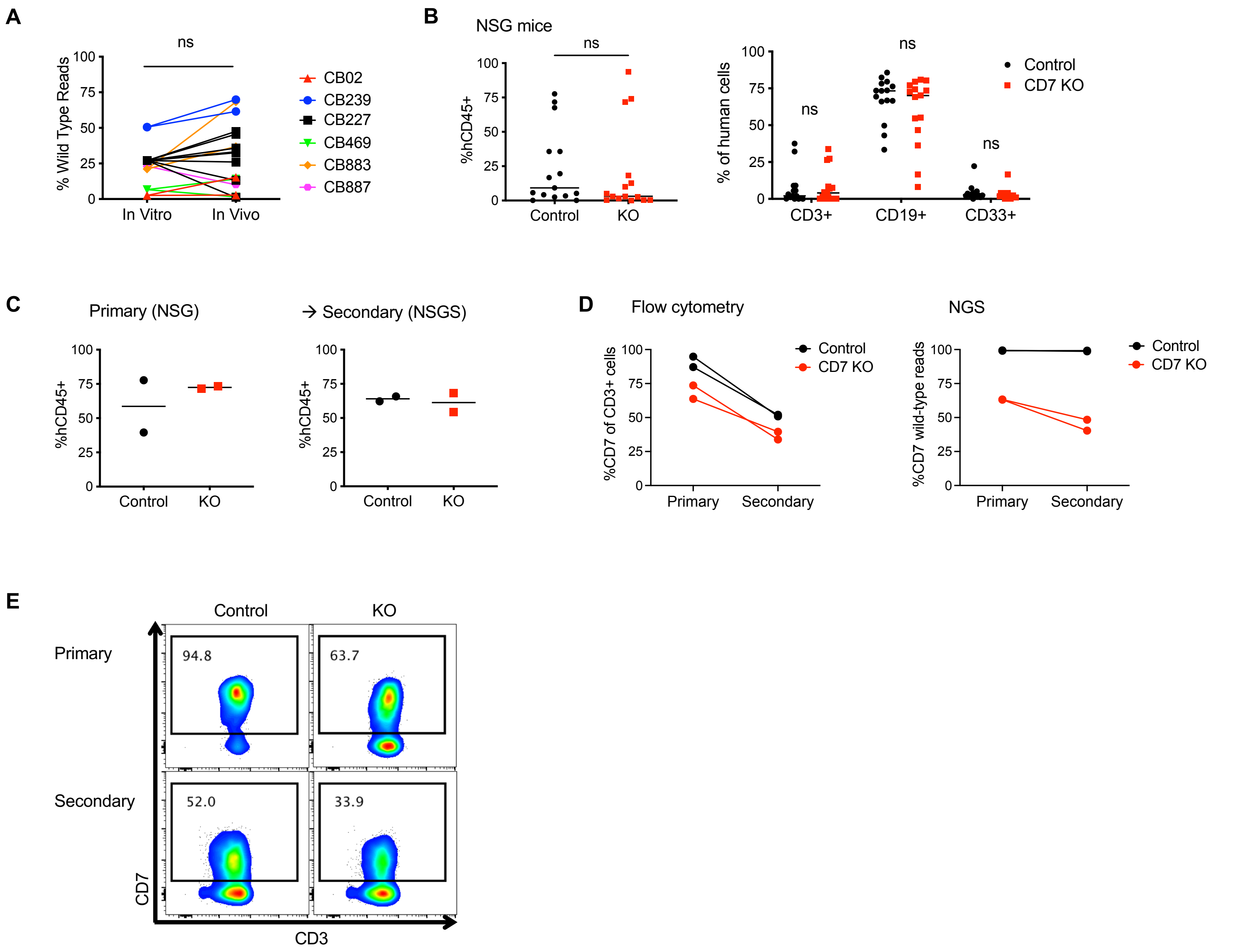

Supplemental Figure 4. CD7 KO HSCs retain long-term engraftment potential. (A) Levels of *CD7* gene mutations remain stable across multiple cord blood donors after 16 weeks of engraftment in NSG and NSGS mice in vivo (n=6 cord blood samples into 18 mice). (B) NSG mice engrafted with control or CD7 KO HSCs show similar engraftment and differentiation in the peripheral blood after 16 weeks (n=15/group). (C-E) Bone marrow from NSG mice were harvested after 17 weeks of primary engraftment and injected intravenously into NSGS mice at a 1:1 ratio. Human engraftment was measured in the secondary transplants at 14 weeks (n=2/group). (C) Secondary transplants from NSG mice into NSGS mice have comparable levels of engraftment for control and CD7 KO HSCs. (D) Secondary transplants continue to exhibit decreased CD7 protein expression by flow cytometry and mutations in the *CD7* gene by next-generation sequencing (NGS). (E) Representative flow plots showing CD7 expression on human T cells derived from control or CD7 KO HSC-engrafted mice in primary and secondary transplanted mice. P-values were determined by paired (A) or unpaired Student's *t*-test (B). ns=non-significant.
